# Medullary Endocannabinoids Contribute to the Differential Resting Baroreflex Sensitivity in Rats with Altered Brain Renin-Angiotensin System Expression

**DOI:** 10.3389/fphys.2016.00207

**Published:** 2016-06-09

**Authors:** Chris L. Schaich, Megan Grabenauer, Brian F. Thomas, Hossam A. Shaltout, Patricia E. Gallagher, Allyn C. Howlett, Debra I. Diz

**Affiliations:** ^1^Department of Physiology and Pharmacology and Hypertension and Vascular Research Center, Wake Forest School of MedicineWinston-Salem, NC, USA; ^2^Analytical Chemistry and Pharmaceutics, RTI InternationalResearch Triangle Park, NC, USA; ^3^Department of Obstetrics and Gynecology, Wake Forest School of MedicineWinston-Salem, NC, USA

**Keywords:** endocannabinoid system, baroreflex sensitivity, NTS, renin-angiotensin system

## Abstract

CB_1_ cannabinoid receptors are expressed on vagal afferent fibers and neurons within the solitary tract nucleus (NTS), providing anatomical evidence for their role in arterial baroreflex modulation. To better understand the relationship between the brain renin-angiotensin system (RAS) and endocannabinoid expression within the NTS, we measured dorsal medullary endocannabinoid tissue content and the effects of CB_1_ receptor blockade at this brain site on cardiac baroreflex sensitivity (BRS) in ASrAOGEN rats with low glial angiotensinogen, normal Sprague-Dawley rats and (mRen2)27 rats with upregulated brain RAS expression. Mass spectrometry revealed higher levels of the endocannabinoid 2-arachidonoylglycerol in (mRen2)27 compared to ASrAOGEN rats (2.70 ± 0.28 vs. 1.17 ± 0.09 ng/mg tissue; *P* < 0.01), while Sprague-Dawley rats had intermediate content (1.85 ± 0.27 ng/mg tissue). Microinjection of the CB_1_receptor antagonist SR141716A (36 pmol) into the NTS did not change cardiac BRS in anesthetized Sprague-Dawley rats (1.04 ± 0.05 ms/mmHg baseline vs. 1.17 ± 0.11 ms/mmHg after 10 min). However, SR141716A in (mRen2)27 rats dose-dependently improved BRS in this strain: 0.36 pmol of SR141716A increased BRS from 0.43 ± 0.03 to 0.71 ± 0.04 ms/mmHg (*P* < 0.001), and 36 pmol of SR141716A increased BRS from 0.47 ± 0.02 to 0.94 ± 0.10 ms/mmHg (*P* < 0.01). In contrast, 0.36 pmol (1.50 ± 0.12 vs. 0.86 ± 0.08 ms/mmHg; *P* < 0.05) and 36 pmol (1.38 ± 0.16 vs. 0.46 ± 0.003 ms/mmHg; *P* < 0.01) of SR141716A significantly reduced BRS in ASrAOGEN rats. These observations reveal differential dose-related effects of the brain endocannabinoid system that influence cardiovagal BRS in animals with genetic alterations in the brain RAS.

## Introduction

Tonic overactivation of the endocannabinoid system is implicated in the maintenance of cardiovascular diseases and its risk factors, including hypertension, atherosclerosis and metabolic syndrome (Pacher et al., 2006; Després, 2007). These adverse conditions are associated with dysfunction of the arterial baroreflex and reduced heart rate variability, themselves indices of general morbidity and mortality (Thayer and Lane, 2007). Baroreflex sensitivity (BRS) for control of heart rate (HR), measured as the bradycardia evoked in response to increases in mean arterial pressure (MAP), is an important indicator of parasympathetic activity and vagus nerve function, and its impairment often precedes the development of cardiovascular disease (Thayer and Lane, 2007).

The G protein-coupled CB_1_ cannabinoid receptor is widely expressed in the central nervous system (CNS) and in peripheral tissues, enabling the activated receptor to regulate a variety of physiological functions including blood pressure and heart rate (Pertwee, 2006). Presynaptic CB_1_ receptors are densely expressed in the solitary tract nucleus (NTS) of the dorsomedial medulla, the primary termination site for baroreceptor afferent neurons relaying sensory information from the periphery, as well as in vagal afferent neurons originating in the nodose ganglion (Mailleux and Vanderhaeghen, 1992; Burdyga et al., 2010). Several studies show that exogenously-applied anandamide, one of two primary endocannabinoid signaling molecules along with 2-arachidonoylglycerol (2-AG), within the NTS modulates baroreflex-evoked suppression of renal sympathetic nerve activity in rats by altering release of the inhibitory neurotransmitter GABA (Seagard et al., 2004; Brozoski et al., 2005). Therefore, the brain endocannabinoid system may influence BRS through the modulation of neurotransmitter release in brain sites regulating effects on the baroreflex.

There is growing evidence for interactions between the endocannabinoid system and the hypertensive actions of the renin-angiotensin system (RAS) (Rozenfeld et al., 2011; Szekeres et al., 2012). Chronic systemic blockade of CB_1_ receptors results in a reduction in blood pressure and improvement in indices of the parasympathetic component of the BRS for control of heart rate in the (mRen2)27 transgenic model of RAS-dependent hypertension (Schaich et al., 2014). In addition, attenuation of BRS following acute injection of Ang II into the NTS of Sprague-Dawley (SD) rats is prevented by prior NTS blockade of CB_1_ receptors (Diz et al., 2015). However, no studies to date have investigated the role of the endocannabinoid system in the NTS of animals with long-term alterations in brain RAS expression.

To test our hypothesis that endocannabinoid content influences cardiac BRS at the level of the NTS in animals with altered brain RAS expression, we studied dorsal medullary endocannabinoid content and function in hemizygous transgenic (mRen2)27 rats, a monogenetic model of Ang II-dependent hypertension in which the mouse *Ren2* renin gene was transfected into the genome of the normotensive Hannover SD rat. These rats have a phenotype of chronic hypertension while conscious with markedly impaired BRS for control of HR compared to SD controls (Bader et al., 1992; Borgonio et al., 2001). In contrast, transgenic ASrAOGEN rats with low brain angiotensinogen, resulting from glial overexpression of an angiotensinogen antisense oligonucleotide against a Hannover SD background, exhibit significantly enhanced resting BRS compared to SD rats (Schinke et al., 1999; Kasper et al., 2005). These strains provide insights into the contribution of the brain RAS and its influence on factors involved in regulation of BRS (Sakima et al., 2007; Diz et al., 2008) and were used to provide functional and biochemical evidence for altered endocannabinoid tone at the level of the NTS.

## Methods

### Animals

Experiments were performed in 15- to 20-week-old male Hannover SD (*N* = 15), and hemizygous transgenic (mRen2)27 (*N* = 18) and TGR(ASrAOGEN)680 (ASrAOGEN; *N* = 17) rats obtained from the Hypertension & Vascular Research Center colony at Wake Forest School of Medicine (Winston-Salem, NC). All microinjection experiments were performed in animals between 16 and 20 weeks of age. Mass spectrometry and reverse transcriptase quantitative polymerase chain reaction (RT-qPCR) were performed in tissue samples from separate groups of 15-week-old animals. Animals were housed two per cage in a humidity- and temperature-controlled room with *ad libitum* access to food (standard chow) and water. The colony was maintained on a 12-h light/dark cycle (lights on at 6:00 a.m.). All experimental procedures were approved by the Institutional Animal Care and Use Committee of Wake Forest School of Medicine.

### Surgical procedures and hemodynamic measures

As previously reported (Sakima et al., 2005, 2007; Arnold et al., 2008, 2009), rats were anesthetized with intraperitoneal combination urethane-chloralose cocktail (750 mg and 35 mg per kg, respectively). Intravenous supplemental doses diluted to 3:7 with saline were given as needed. Polyethylene catheters (polyethylene-10 and −50 tubing; Becton, Dickinson and Company, Franklin Lakes, NJ) were inserted into the femoral artery and vein, with the venous catheter proximal end positioned near the right atrium, for measurement of cardiovascular parameters and administration of drugs, respectively. Rats were then placed in a stereotaxic frame with the head tilted at a 45° downward angle for surgical exposure of the dorsal medulla oblongata by incision of the atlanto-occipital lobe. Rats breathed a mixture of 30% oxygen and 70% room air with body temperature maintained at 37 ± 1°C. An equilibration period of approximately 30 min was allowed after surgical procedures before baseline BRS measurements began. A data acquisition system (BIOPAC Systems Inc.; Acq*Knowledge* software v.3.8.1; Goleta, CA) recorded and digitized pulsatile arterial pressure (AP) and mean AP (MAP). Heart rate (HR) was calculated from the AP wave as previously reported (Sakima et al., 2005, 2007). Baseline measures of baroreflex sensitivity (BRS) were established by bolus intravenous injection of 3 doses of phenylephrine (2, 5, and 10 μg/kg in 0.9% NaCl) administered in random order to determine the bradycardic BRS in response to increases in AP. Bolus dose determinations were used because this method is more sensitive to alterations in the parasympathetic baroreflex relative to infusion determinations (Korner, 1995). A minimum of 15 min was allowed after baseline measurements before microinjections. Maximum MAP responses (ΔMAP, mmHg) and the associated reflex changes in HR (ΔHR, bpm) were recorded after each dose of phenylephrine and compared to the 30-s average during the period immediately preceding each phenylephrine infusion. ΔHR was converted to changes in pulse interval (ΔPI, ms) by the formula 60,000/HR. Bradycardic BRS was defined for each animal as the slope of the relationship between ΔMAP and corresponding ΔPI generated independently from the 3 doses of phenylephrine, as previously reported (Sakima et al., 2005, 2007; Arnold et al., 2008, 2009). A minimum of 5 min elapsed between each phenylephrine infusion to allow normalization of MAP and HR. BRS testing was repeated within 10 and 60 min of SR141716A microinjection so that each animal served as its own control, with testing completed within 15 min after starting. In addition, a single intravenous bolus infusion of phenylbiguanide (10 μg/kg in 0.9% NaCl) was administered following phenylephrine testing before and after NTS microinjections to assess depression in HR due to activation of the chemosensitive vagal afferent fibers.

Spontaneous BRS was also calculated by spectral analysis of the AP time and frequency domains using the Nevrokard SA-BRS software (Medistar, Ljubljana, Slovenia), as previously described (Shaltout and Abdel-Rahman, 2005). Spontaneous BRS was determined from a minimum of 10 min of AP recordings obtained during baseline recording and again within 10 min of SR141716A microinjection, and calculated in the time (Sequence [Seq] Up, Seq Down, and Seq All; in units of milliseconds per mmHg) and frequency (low-frequency [LF] and high-frequency [HF] α indices) domains.

### NTS microinjections

Multi-barreled glass pipettes (outer diameter of 30–50 μm) were used as previously described (Sakima et al., 2005, 2007; Arnold et al., 2008, 2009) for NTS microinjections. SR141716A (obtained from the National Institute on Drug Abuse; 0.36 or 36 pmol [0.167 and 16.7 ng, respectively] in a 120 nL volume of 10% DMSO in artificial cerebrospinal fluid titrated to pH 7.4) or vehicle (120 nL) was bilaterally microinjected via pressure from a hand-held syringe (Becton, Dickinson and Company) into the NTS [0.4 mm rostral, 0.4 mm lateral to the calamus scriptorius [caudal tip of the area postrema] and 0.4 mm below the dorsal surface of the medulla) connected via polyethylene-50 tubing to a glass micropipette. Air pressure was generated by pushing on the syringe to displace the desired amount of SR141716A from the micropipette into the NTS, visualized by movement of the fluid meniscus across the calibration line of the pipette barrel as described (Sakima et al., 2005, 2007). The doses and volume of SR141716A were comparable to previous NTS microinjection studies in which the drug completely reversed or prevented alteration of BRS by microinjection of a cannabinoid receptor agonist (Schaich et al., 2011). At the end of each experiment, animals were euthanized by intravenous overdose of urethane-chloralose anesthesia and decapitated. Brains were removed and frozen on dry ice for verification of the microinjection site in serial cryostat sections (30 μm) of the frozen medulla (Figure S1). Only data from injections within the medial NTS at rostro-caudal level −13.3 to 14.0 mm caudal to bregma are reflected in the analysis. The accuracy rate for microinjections was > 90%.

### Mass spectrometry detection of anandamide and 2-AG

Medullary tissue was obtained from separate groups of naïve 15-week-old SD, (mRen2)27 and ASrAOGEN rats (*n* = 4–5 each strain) not used in microinjection experiments. Animals were decapitated while conscious to avoid potential confounding effects of anesthesia on brain endocannabinoid levels. Brains were removed and frozen on dry ice for excision of 3-mm^3^ dorsal medullary sections. The sections were obtained from 1.5 mm anterior to 1.5 mm posterior to the usual placement of the microinjection pipette, corresponding with the expected injectate spread within the NTS (Campagnole-Santos et al., 1988) and including portions of the area postrema, dorsal motor nucleus, and nucleus gracilis. For the analysis of the two major endocannabinoids anandamide and 2-AG, an extraction technique was developed for rat medulla tissue. Optimal extraction conditions for brain tissue were obtained using high performance liquid chromatography (HPLC)-grade acetonitrile and tissue homogenization with stainless steel beads (2.3 mm, BioSpec Products, Bartlesville, OK) that reduced enzymatic degradation and attenuated 2-AG isomerization. Tissue homogenates were fortified with isotopically-labeled internal standards for 2-AG and anandamide 2-[^2^H_5_]2-AG (AG-d5) and [^2^H_4_]anandamide (AEA-d4) on a per-mg basis for sample integrity and robustness of the method. The chromatography solvents were Honeywell B&J HPLC-grade ammonium acetate and acetic acid–double distilled (Sigma-Aldrich). Stock solutions of anandamide and 2-AG and their deuterated analogs were freshly prepared and stored as recommended by the manufacturer. The study samples, quality control samples, and standards (targeted analysis) were processed using automated liquid handling to ensure the reproducibility and consistency of the sample preparation method.

A Waters Acuity ultraperformance liquid chromatography (UPLC) system (Waters, Milford, MA) equipped with an Acquity UPLC BEH C18 1.7 μm 2.1 × 50 mm column was utilized for the separation of endocannabinoids prior to quantitative analysis by selected reaction monitoring (LC-MS/MS). An API-5000 triple quadrupole mass spectrometer (Applied Biosystems/Sciex, Carlsbad, CA) was coupled to the UPLC for quantitative analysis, and operating parameters were optimized for anandamide and 2-AG. Calibration curves spanning the range of quantitation were produced through least-squares linear regression analysis of response (analyte integrated area/internal standard integrated area) vs. concentration, and may be weighted to improve fit, accuracy and precision. Sample content of anandamide or 2-AG is expressed as ng analyte per mg tissue wet weight.

### Quantification of CB_1_, CB_2_, and CRIP1a mRNA

RT-qPCR was used to measure mRNA levels of various effector components of the brain endocannabinoid system, including CB_1_ and CB_2_ receptor, and CRIP1a (Niehaus et al., 2007) in dorsal medullary tissue from the same naïve 15-week-old SD, (mRen2)27 and ASrAOGEN rats (n = 4-6 each strain) used for detection of medullary endocannabinoids. Brains were removed and frozen on dry ice as described above. Total RNA was extracted from brain sections using TRIZOL reagent (Invitrogen, Carlsbad, CA) and was assessed for concentration and stability using an Agilent 2100 Bioanalyzer with an RNA 6000 nano LabChip (Agilent Technologies, Palo Alto, CA). Total RNA (1 μg) was reverse transcribed using AMV reverse transcriptase in a 20-μL reaction mixture containing deoxyribonucleotides, random hexamers, and RNase inhibitor in reverse-transcriptase buffer, as described previously (Sakima et al., 2007; Arnold et al., 2008, 2009). The reaction was terminated by heating the reverse transcriptase reaction product at 95°C. For RT-qPCR, 2 μL of resultant cDNA were added to TaqMan Universal PCR Master Mix (Applied Biosystems) with the appropriate gene-specific primer/probe set for CB_1_ and CB_2_ receptors, and CRIP1a (Applied Biosystems), and amplification was performed on an ABI 7000 Sequence Detection System. The mixtures were heated for 2 min at 50° C, 10 min at 95°C, followed by 40 cycles at 95°C for 15 s and 60°C for 60 s. All reactions were performed in triplicate. 18S ribosomal RNA, amplified using the TaqMan Ribosomal RNA Control Kit (Applied Biosystems) served as the internal control. Results were quantified as Ct values, in which Ct is the threshold cycle of PCR at which an amplified product is first detected, and was defined as relative gene expression (ratio of target:control).

### Analysis of data

Values are presented as mean ± SEM. Comparisons of changes in BRS over time in response to SR141716A or vehicle in the different strains were assessed using a repeated measures two-way ANOVA. Where appropriate on the basis of the two-way ANOVA, one-way ANOVA and post-hoc Bonferroni multiple comparisons elucidated further differences between strains or time points. Multiple regression analysis was performed on BRS scatterplots to determine slopes of fit lines. The criterion for statistical significance was *P* < 0.05. Statistical tests were performed using Prism 5.0 (GraphPad Software, San Diego, CA).

## Results

### Baseline BRS for control of HR in response to increases in MAP in SD, (mRen2)27 and ASrAOGEN rats

The pooled bradycardic BRS for control of HR at baseline for ASrAOGEN rats was significantly higher compared to SD rats (1.43 ± 0.07 vs. 1.03 ± 0.06 ms/mmHg; *P* < 0.001; *n* = 11 and 10, respectively; Figure S2), while baseline BRS in (mRen2)27 rats was significantly lower than in SD rats (0.44 ± 0.02 ms/mmHg; *P* < 0.001; *n* = 13; Figure S2). These observations are fully consistent with previous studies from our laboratory (Sakima et al., 2007; Diz et al., 2008). Furthermore, there were no differences in baseline BRS values of SD, (mRen2)27 or ASrAOGEN rat treatment groups receiving vehicle or various doses of SR141716A (Figure S3).

### Effect of CB_1_ antagonist on BRS for control of HR in SD, (mRen2)27 and ASrAOGEN rats

**Figure 1:**
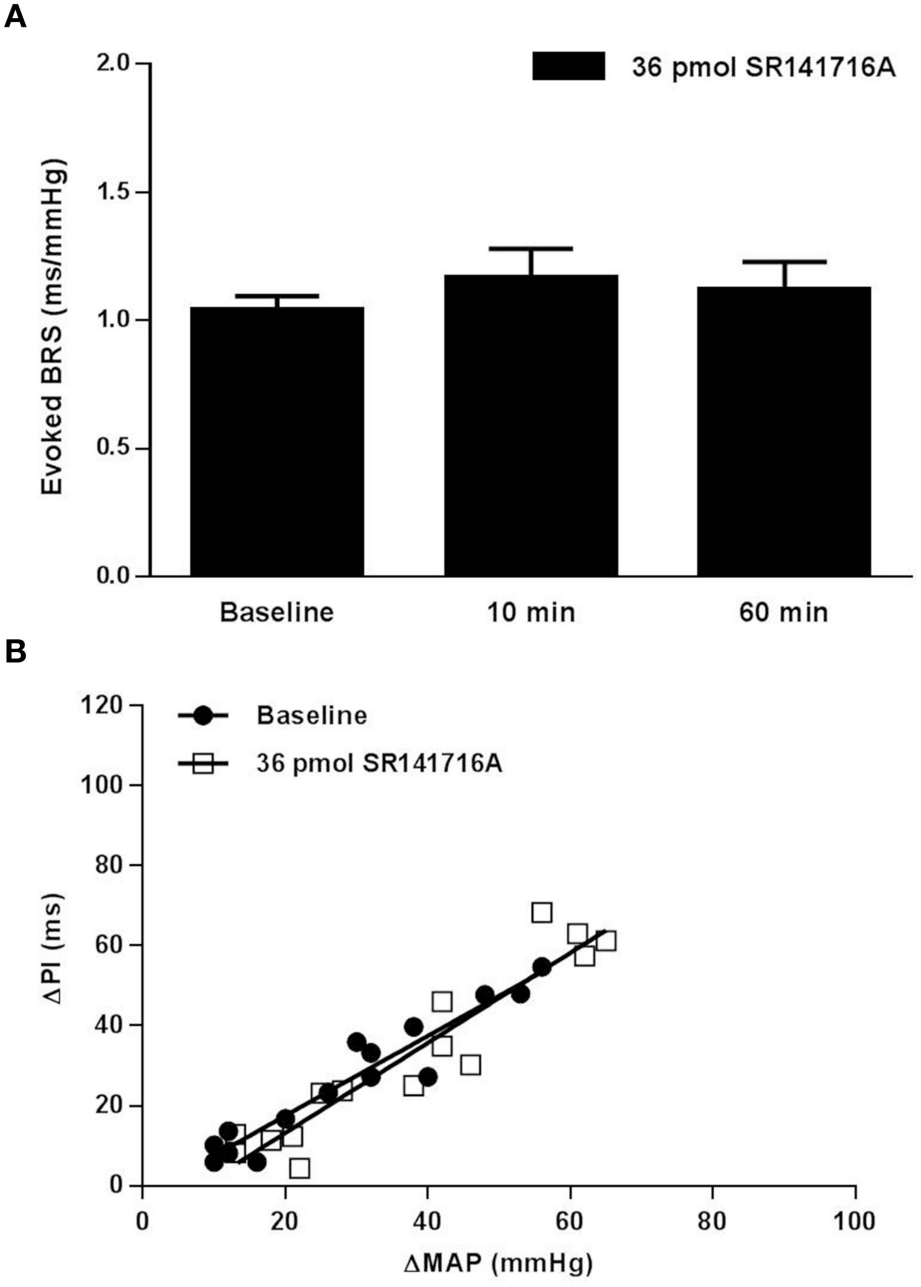
**Effect of NTS microinjection of CB_1_ receptor antagonist SR141716A on BRS for control of HR evoked by phenylephrine in SD rats. (A)** NTS microinjection of 36 pmol of SR141716A in SD rats did not significantly change BRS after 10 min, nor after 60 min in the same animals (*n* = 5). Note: only the 36-pmol dose of SR141716A was tested in SD rats. **(B)** The slope of the relationship between the increases in MAP produced by phenylephrine and the corresponding reflex bradycardia (ΔPI) does not show change from baseline in the linear regression slope 10 min following NTS microinjection of 36 pmol of SR141716A (1.00 ± 0.08 ms/mmHg baseline; 1.13 ± 0.11 ms/mmHg after 10 min; *R*^2^ = 0.84 to 0.92 for pooled data).

**Figure 2:**
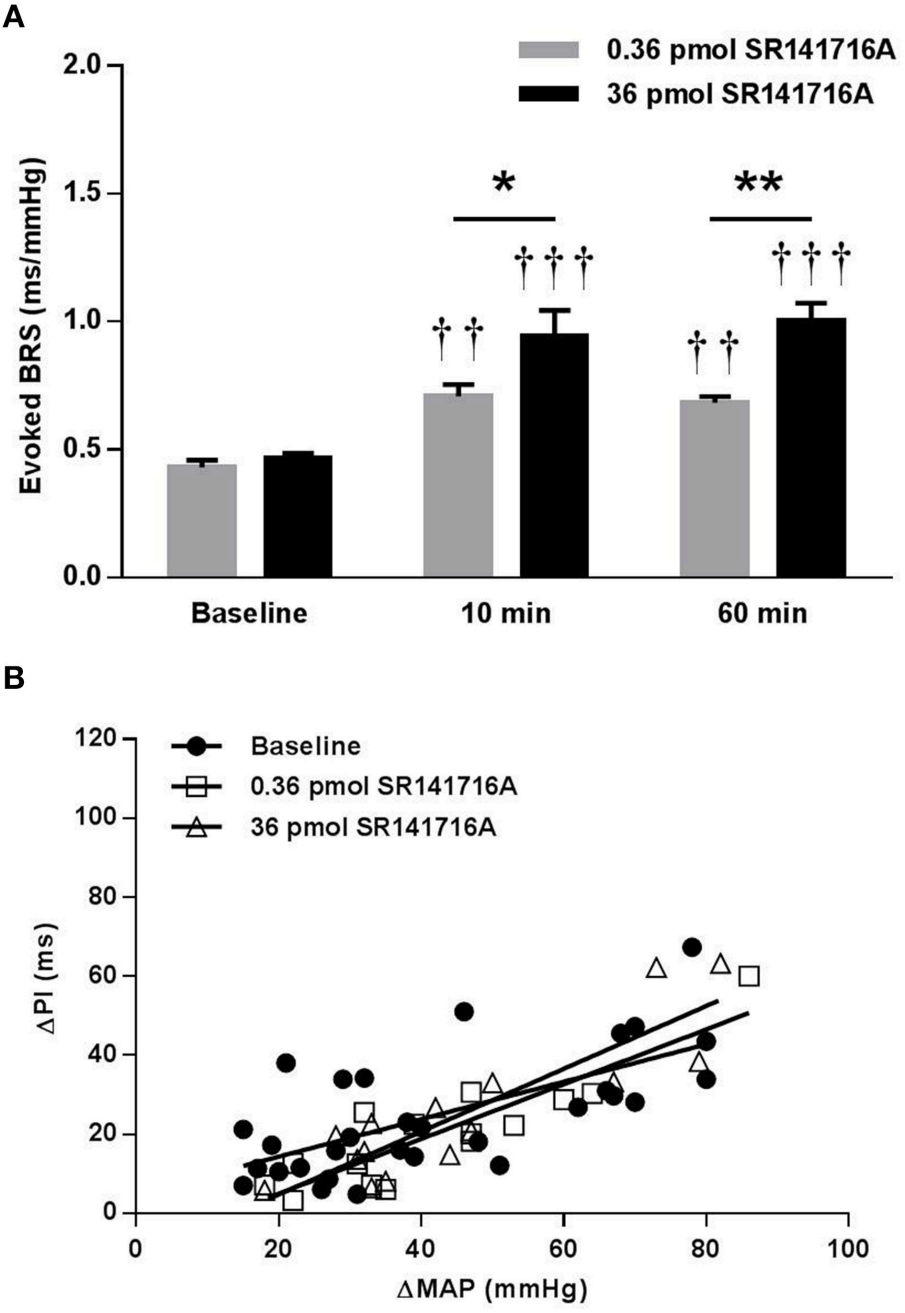
**Effect of NTS microinjection of SR141716A on BRS for control of HR evoked by phenylephrine in (mRen2)27 rats. (A)** In (mRen2)27 rats, NTS microinjection of 0.36- (*n* = 5) and 36-pmol (*n* = 5) of SR141716A significantly improved BRS after 10 and 60 min. **(B)** The 0.36- and 36- pmol doses produced graded, though not statistically significant, increases in the slope of the regression line in (mRen2)27 rats (0.47 ± 0.10 ms/mmHg baseline; 0.69 ± 0.10 ms/mmHg 10 min after 0.36 pmol of SR141716A; 0.79 ± 0.12 ms/mmHg 10 min after 36 pmol SR141716A; *R*^2^ = 0.44 to 0.79 for pooled data). ^*^P < 0.05; ^**^P < 0.01; ^††^*P* < 0.01 vs. baseline; ^†††^*P* < 0.001 vs. baseline.

In SD rats, NTS microinjection of 36 pmol of SR141716A had no effect on BRS for control of HR in response to increases in MAP produced by phenylephrine at 10 or 60 min after microinjection (Figure 1). The lower 0.36 pmol dose of SR141716A was not tested in SD rats due to the lack of effect by the 36 pmol dose. However, microinjection of 0.36- and 36 pmol of SR141716A into the NTS of (mRen2)27 rats significantly and dose-dependently increased BRS by 65% (*P* < 0.01 vs. baseline) and 100% (*P* < 0.001 vs. baseline; *P* < 0.05 vs. 0.36-pmol dose), respectively, after 10 min (Figures 2A,B). Timecourse experiments showed that the BRS-enhancing effect of each dose in (mRen2)27 rats was maintained at 60 min post-microinjection (Figure 2A).

**Figure 3:**
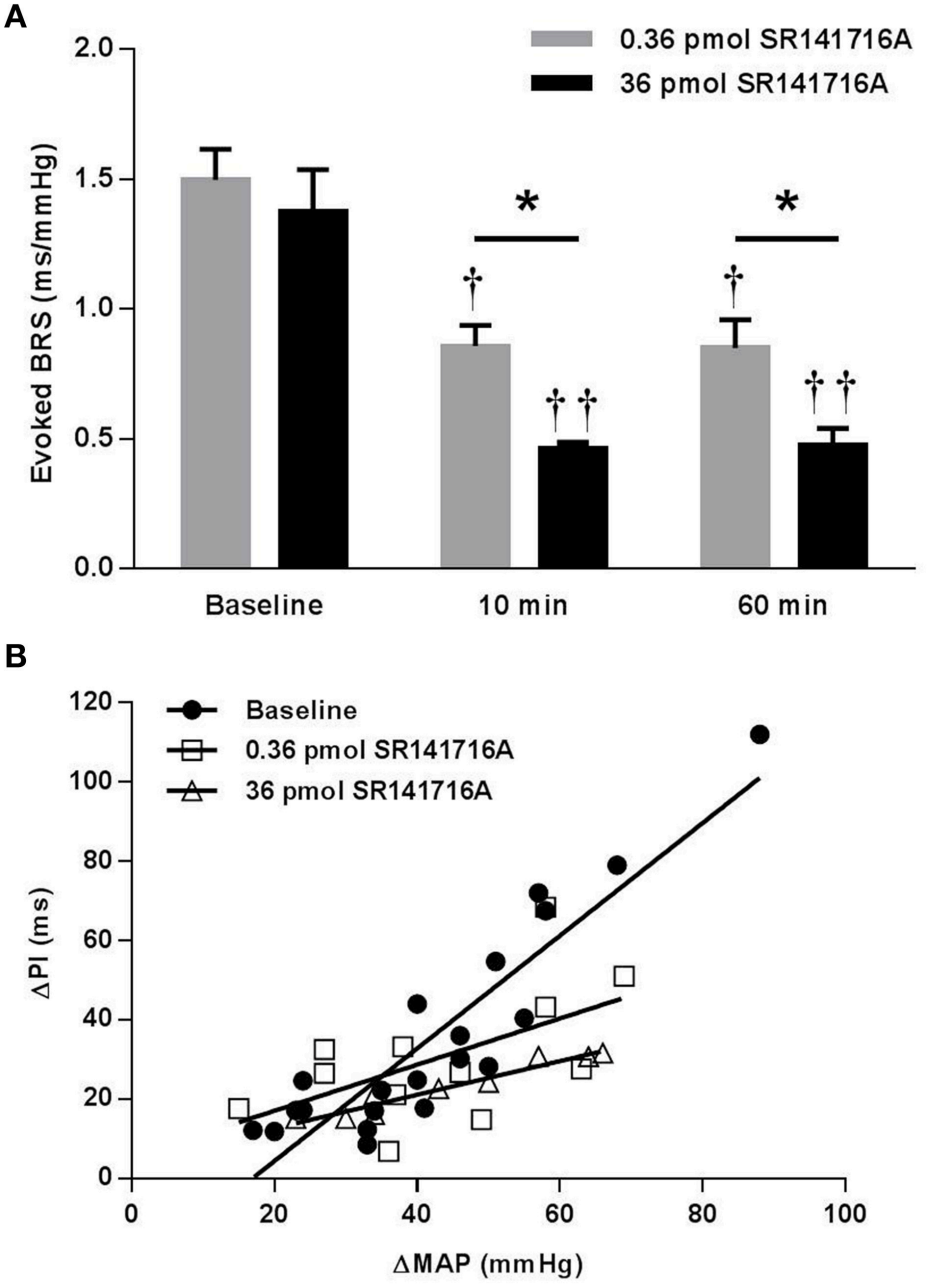
**Effect of NTS microinjection of SR141716A on BRS for control of HR evoked by phenylephrine in ASrAOGEN rats. (A)** In ASrAOGEN rats, NTS microinjection of 0.36- (*n* = 4) and 36-pmol (*n* = 3) of SR141716A significantly attenuated BRS after 10 and 60 min. **(B)** The 0.36- and 36- pmol doses of SR141716A produced equivalent, statistically significant reductions in the slope of the regression line in ASrAOGEN rats (1.42 ± 0.15 ms/mmHg baseline; 0.58 ± 0.27 ms/mmHg 10 min after 0.36 pmol of SR141716A; 0.42 ± 0.04 ms/mmHg 10 min after 36 pmol SR141716A; *P* < 0.001; *R*^2^ = 0.32 to 0.93 for pooled data). ^*^P < 0.05; ^†^*P* < 0.05 vs. baseline; ^††^*P* < 0.01 vs. baseline.

In contrast to the potentiating effect found in (mRen2)27 rats, NTS microinjection of 0.36 pmol SR141716A in ASrAOGEN rats significantly reduced cardiac BRS by 43% (*P* < 0.05 vs. baseline), while 36 pmol of SR141716A reduced BRS by 67% after 10 min (*P* < 0.01 vs. baseline; *P* < 0.05 vs. 0.36-pmol dose; Figures 3A,B). As in (mRen2)27 rats, timecourse experiments showed that the attenuating effect of each dose on BRS was maintained at 60 min after microinjection (Figure 3A).

Spontaneous BRS (sBRS) values obtained by spectral analysis of the AP wave following administration of SR141716A in (mRen2)27 and ASrAOGEN rats are consistent with evoked BRS measurements. In (mRen2)27 rats SR141716A significantly improved sBRS (Seq All), including the parasympathetic sBRS (Seq Up), after both 0.36 and 36 pmol microinjections (*P* < 0.05; Table 1), whereas in ASrAOGEN rats 36 pmol of SR141716A significantly attenuated Seq All and Seq Up (*P* < 0.05; Table 1). There was no effect of SR141716A microinjection on the sympathetic sBRS (Seq Down) in either transgenic strain, nor were there any changes in sBRS in SD rats (Table 1).

**Table 1:** **Effects of acute NTS microinjection of SR141716A on indices of spontaneous BRS in SD, (mRen2)27 and ASrAOGEN rats**.

**Group**	**Seq Up (ms/mmHg)**	**Seq Down (ms/mmHg)**	**Seq All (ms/mmHg)**	**LFα**	**HFα**
**SD 36 pmol**					
Baseline	1.58 ± 0.30	1.64 ± 0.34	1.61 ± 0.33	1.24 ± 0.10	1.72 ± 0.45
After SR141716A	2.21 ± 0.45	1.70 ± 0.38	2.15 ± 0.43	1.69 ± 0.46	2.72 ± 0.59
**(mRen2)27 0.36 pmol**					
Baseline	0.86 ± 0.21	0.88 ± 0.19	0.88 ± 0.17	1.11 ± 0.23	0.75 ± 0.83
After SR141716A	1.01 ± 0.28^*^	1.04 ± 0.20	1.04 ± 0.25^*^	1.37 ± 0.62	0.83 ± 0.30
**(mRen2)27 36 pmol**					
Baseline	0.77 ± 0.14	0.79 ± 0.10	0.78 ± 0.10	0.76 ± 0.15	0.66 ± 0.16
After SR141716A	1.24 ± 0.27^*^	1.38 ± 0.42	1.25 ± 0.21^*^	0.79 ± 0.10	0.91 ± 0.16
**ASrAOGEN 0.36 pmol**					
Baseline	0.99 ± 0.15	1.00 ± 0.14	0.98 ± 0.13	1.07 ± 0.60	1.02 ± 0.20
After SR141716A	0.56 ± 0.10	0.69 ± 0.10	0.63 ± 0.09	0.65 ± 0.24	0.44 ± 0.09^*^
**ASrAOGEN 36 pmol**					
Baseline	0.97 ± 0.08	0.93 ± 0.11	0.97 ± 0.02	1.12 ± 0.04	0.89 ± 0.09
After SR141716A	0.68 ± 0.06^*^	0.63 ± 0.17	0.69 ± 0.08^*^	0.78 ± 0.25	0.64 ± 0.06

*Values are mean ± SEM and represent indices of spontaneous BRS measured in the time (Seq Up, Seq Down, Seq All) and frequency (LFα and HFα) domains before and within 10 min of NTS microinjection of SR141716A*.

* *P < 0.05 vs. respective baseline; n = 3–5 all groups*.

### MAP and HR responses to NTS microinjection of CB_1_ antagonist

There were no significant differences in baseline MAP or HR within groups of anesthetized SD, (mRen2)27 or ASrAOGEN rats receiving vehicle or SR141716A microinjections, nor were there significant differences between pooled baseline HR of SD and (mRen2)27 rats receiving vehicle or SR141716A (Table 2 and Table S1; Figure S4). However, as reported previously, the pooled baseline MAP of anesthetized ASrAOGEN rats was significantly higher compared to SD rats (115 ± 4 vs. 94 ± 2 mmHg, respectively; *P* < 0.01; Figure S4). The pooled baseline HR was also significantly higher in ASrAOGEN rats compared to SD and (mRen2)27 rats (347 ± 7 vs. 313 ± 8 and 312 ± 6 bpm, respectively; *P* < 0.01; Figure S3). The pooled baseline MAP of anesthetized (mRen2)27 rats (108 ± 4 ms/mmHg) was also significantly higher compared to SD rats (*P* < 0.05; Figure S4).

Acute NTS microinjection of either 0.36 or 36 pmol of SR141716A did not significantly change resting MAP in SD or ASrAOGEN rats, nor HR in SD, (mRen2)27 or ASrAOGEN rats at the time of reflex testing 10 and 60 min after microinjection. While 0.36 pmol of SR141716A did not have a significant effect on resting MAP in (mRen2)27 rats over the duration of testing, the 36 pmol dose produced a modest yet statistically significant decrease in MAP at 10 and 60 min post-microinjection (*P* < 0.001 vs. baseline; Table 2). There were no significant effects of NTS microinjection of vehicle on MAP or HR in any strain (Table S1). Differences in MAP or HR within groups of transgenic animals from SD rats at individual timepoints are noted in Table 2 and Table S1.

**Table 2:** **Values of MAP and HR in response to NTS microinjection of SR141716A**.

**Group**	*****N*****	**MAP (mmHg)**	**HR (bpm)**
**SD 36 pmol SR141716A**	5		
Baseline		94 ± 3	297 ± 6
Values at 10 min		90 ± 3	280 ± 9
Values at 60 min		91 ± 4	291 ± 11
**(mRen2)27 0.36 pmol SR141716A**	5		
Baseline		103 ± 5	319 ± 5
Values at 10 min		99 ± 6	308 ± 5
Values at 60 min		96 ± 4	326 ± 9^*^
**(mRen2)27 36 pmol SR141716A**	5		
Baseline		119 ± 6^#^	316 ± 5
Values at 10 min		109 ± 7^‡^	318 ± 10^*^
Values at 60 min		105 ± 7^‡^	325 ± 10^*^
**ASrAOGEN 0.36 pmol SR141716A**	4		
Baseline		115 ± 7^*^	347 ± 12^#^
Values at 10 min		113 ± 8^#^	361 ± 14^§^
Values at 60 min		104 ± 4	360 ± 5^§^
**ASrAOGEN 36 pmol SR141716A**	3		
Baseline		107 ± 5	335 ± 7
Values at 10 min		104 ± 7	344 ± 16^#^
Values at 60 min		98 ± 6	345 ± 15^#^

*Values are mean ± SEM and represent baseline MAP and HR and values at 10 or 60 min after the initial SR141716A NTS microinjection; N = number of animals*.

‡ *P < 0.001 vs. baseline*.

* *P < 0.05 vs. SD*;

# *P < 0.01*;

§ *P < 0.001*.

Microinjection of 36 pmol of SR141716A yielded a modest yet statistically significant potentiation of intravenous phenylephrine-induced MAP increases in SD rats after 10 and 60 min (*P* < 0.05). However, there were no differences in MAP responsiveness to phenylephrine injections among groups of (mRen2)27 or ASrAOGEN rats receiving various doses of SR141716A (Figure S5).

Depressor and bradycardic responses to cardiac chemosensitive vagal fiber activation induced by intravenous phenylbiguanide in SD, (mRen2)27 and ASrAOGEN rats were not altered by NTS microinjection of SR141716 (Figure S6).

### Dorsal medullary 2-AG and anandamide content in SD, (mREN2)27 and ASrAOGEN rats

**Figure 4:**
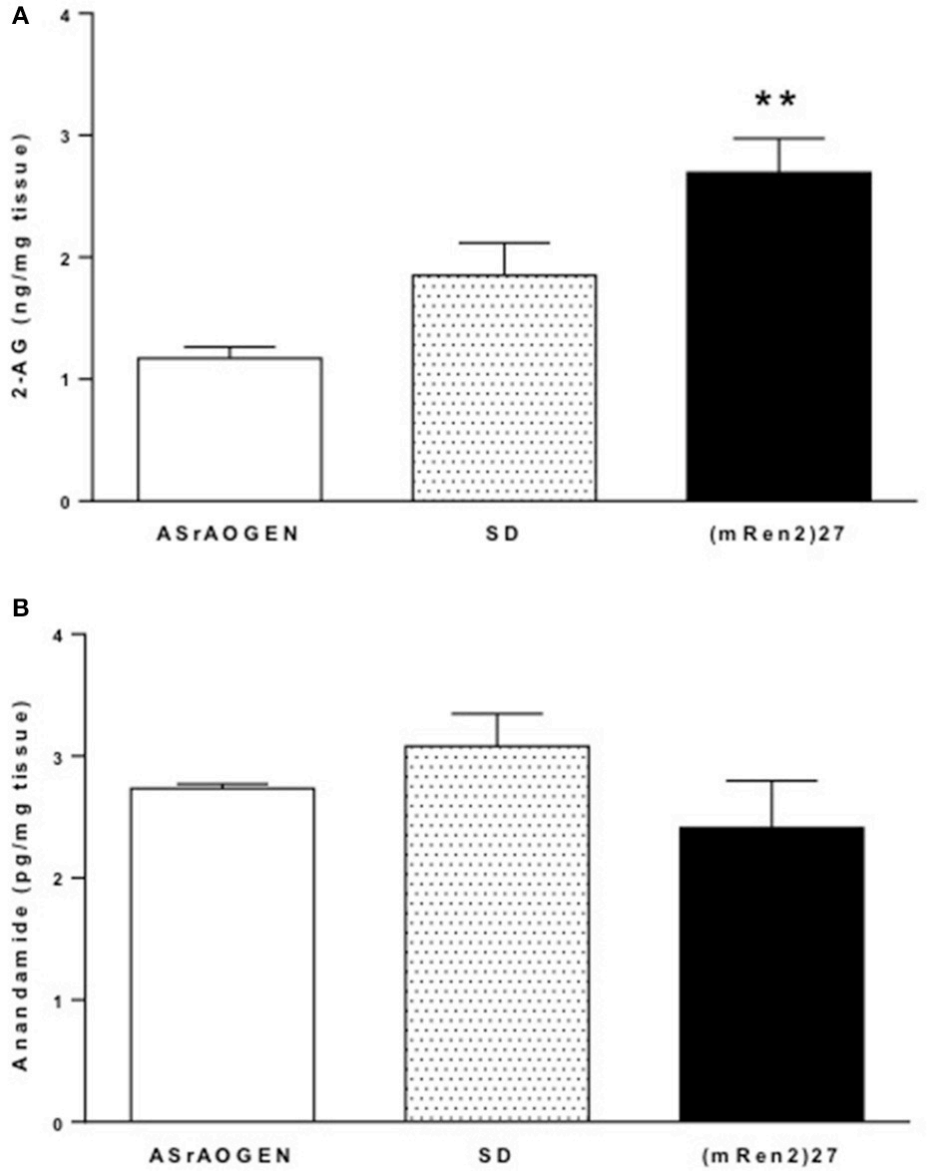
**2-AG and anandamide content in dorsal medulla of SD, (mRen2)27 and ASrAOGEN rats. (A)** Mass spectrometry revealed levels of 2-AG were significantly higher in the dorsal medullary tissue of 15-week-old (mRen2)27 rats (*n* = 5) relative to ASrAOGEN rats (*n* = 4). **(B)** Anandamide was detected at levels 1000-fold lower than 2-AG in the same tissue samples, and there were no significant differences among strains. ^**^P < 0.01 vs. ASrAOGEN.

Mass spectrometry revealed that concentrations of 2-AG were highest in dorsal medulla of (mRen2)27 rats (2.70 ± 0.28 ng/mg tissue vs. 1.17 ± 0.09 ng/mg tissue in ASrAOGEN rats; *P* < 0.01; *n* = 4–5; Figure 4A). SD rats had intermediate medullary 2-AG content (1.85 ± 0.27 ng/mg tissue; *n* = 5), falling short of statistical significance compared to (mRen2)27 rats (*P* = 0.069) and ASrAOGEN rats (*P* = 0.052). Dorsal medullary anandamide was found in levels 1000-fold lower than 2-AG and there were no significant differences among rat strains (Figure 4B).

### CB_1_, CB_2_ and CRIP1a mRNA in SD, (mREN2)27 and ASrAOGEN rats

**Figure 5:**
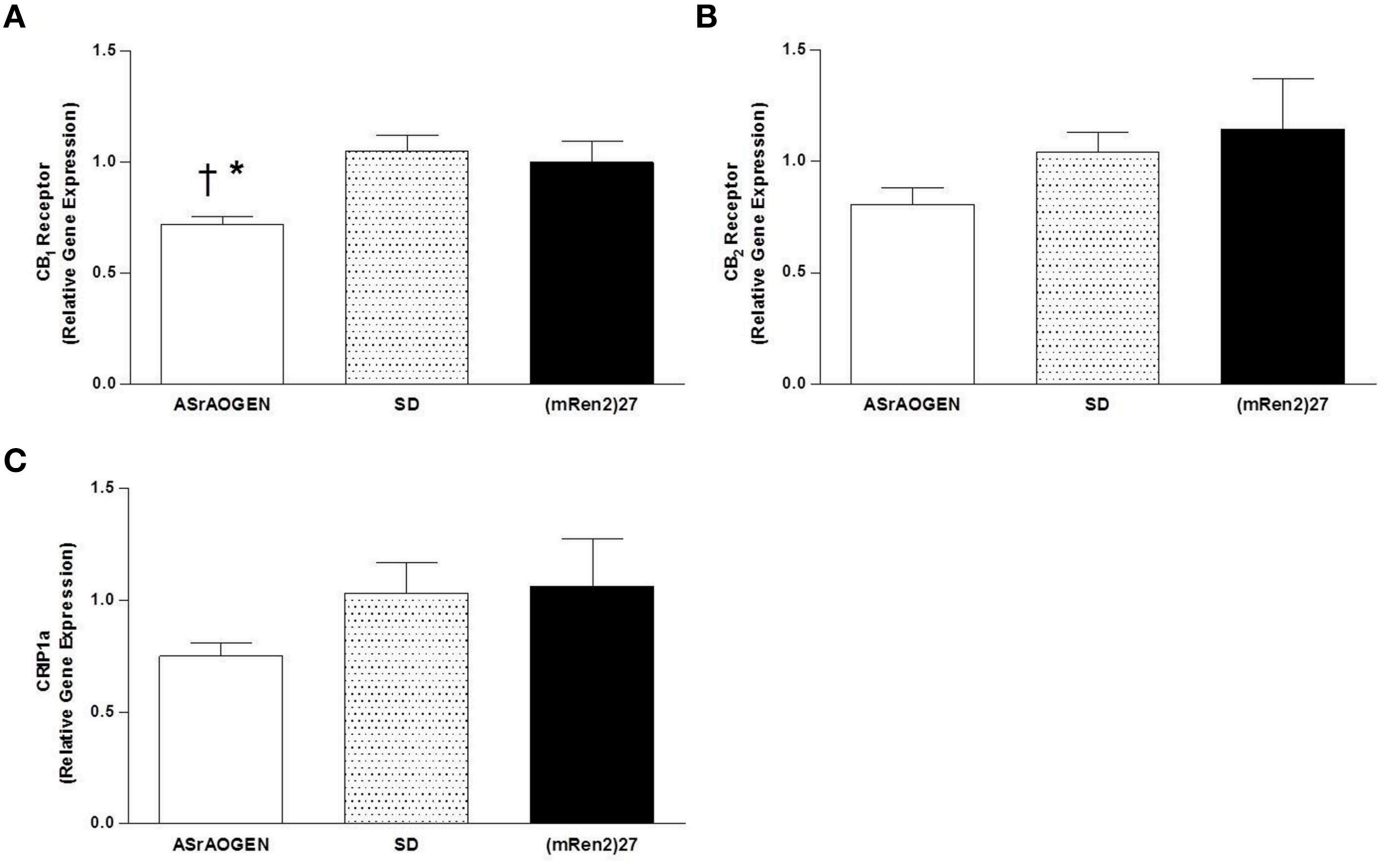
**CB_1_, CB_2_ and CRIP1a mRNA in the dorsal medulla of SD, (mRen2)27 and ASrAOGEN rats. (A)** Relative gene expression of CB_1_ receptor was lower in the dorsal medullary tissue of 15-week-old ASrAOGEN rats (*n* = 6) compared to SD (*n* = 5) and (mRen2)27 (*n* = 4) rats. **(B,C)** No significant differences in relative gene expression of CB_2_ receptor **(B)** or CRIP1a **(C)** were found among strains. ^*^P < 0.05 vs. (mRen2)27; ^†^*P* < 0.01 vs. SD.

Relative gene expression of CB_1_ and CB_2_ receptors, and the endocannabinoid regulatory protein CRIP1a was measured in dorsal medullary tissue of naïve SD (*n* = 5), (mRen2)27 (*n* = 4), and ASrAOGEN (*n* = 6) rats at 15 weeks of age (Figure 5). CB_1_ receptor mRNA was approximately 0.25-fold lower in the dorsal medulla of ASrAOGEN rats relative to SD (*P* < 0.01) and (mRen2)27 (*P* < 0.05) rats (Figure 5A). There were no differences in relative mRNA levels of CB_2_ receptor or CRIP1a among the same rat groups (Figures 5B,C).

## Discussion

In this study, we report for the first time the endocannabinoid content and relative receptor mRNA levels associated with the endocannabinoid system are differentially expressed in the dorsal medulla of (mRen2)27 and ASrAOGEN rats compared to SD rats. We also determined the effects of CB_1_ cannabinoid receptor blockade by SR141716A at the level of the NTS on cardiac BRS in response to phenylephrine-evoked increases in MAP in anesthetized rats with differential brain RAS expression profiles. The improvement in BRS in response to SR141716A in (mRen2)27 rats with higher 2-AG content in the dorsal medulla suggests that higher brain Ang II (Senanayake et al., 1994) may contribute to upregulation of 2-AG release resulting in the blunted baseline BRS in this strain. Our findings are consistent with the finding that the effects of Ang II in the NTS to attenuate BRS are blocked by a CB_1_ receptor antagonist (Diz et al., 2015). In ASrAOGEN rats, however, medullary 2-AG content was significantly lower compared to (mRen2)27 rats, and CB_1_ receptor mRNA was significantly lower relative to SD and (mRen2)27 rats. Therefore, the BRS-suppressing effect of SR141716A in the NTS of ASrAOGEN rats suggests differential regulatory functions of the brain endocannabinoid system in rats with high or low brain RAS expression.

Our present study presents evidence for a direct action of endocannabinoids to modulate baroreflex function in transgenic rats over- or underexpressing components of the brain RAS. Microinjection of the selective CB_1_ receptor antagonist SR141716A (0.36 and 36 pmol) into the NTS differentially altered BRS measured as the bradycardic response to phenylephrine-evoked increases in AP in (mRen2)27 rats with Ang II-dependent hypertension and in glial Aogen-deficient ASrAOGEN rats. In (mRen2)27 rats, the 0.36-pmol dose of SR141716A significantly improved BRS, and the 36-pmol dose improved BRS from baseline to a greater degree, indicating a dose-response relationship. In fact, the 36-pmol dose improved BRS in these animals to ≈1.05 ms/mmHg, a level comparable with baseline BRS of SD rats and suggesting normalization of cardiac BRS in (mRen2)27 rats (Schaich et al., 2014). In ASrAOGEN rats, however, SR141716A significantly impaired BRS to ≈0.46 ms/mmHg, a level often observed in hypertension and comparable to baseline BRS of (mRen2)27 rats (Diz et al., 2008). Although it is difficult to determine whether CB_1_ receptor blockade affects resting MAP in anesthetized ASrAOGEN rats due to a small group size, a significant decrease in BRS was clearly detected. The differential BRS responses to CB_1_ receptor antagonism in the NTS of these strains with altered brain endocannabinoid content supports the prior observations of differential baroreflex-evoked responses to exogenously applied CB_1_ agonists in hypertensive vs. normotensive animals that may involve contrasting effects on amino acid transmitter systems (Brozoski et al., 2009). Thus, data are consistent with the interpretation that the medullary endocannabinoid system exerts a tonic influence over baroreflex function via NTS CB_1_ receptors which may contribute to suppressed baseline cardiovagal BRS in (mRen2)27 rats and enhanced baseline BRS in ASrAOGEN rats. Future studies are required to determine the mechanisms underlying these effects.

Unlike in the transgenic animals, there was no effect of CB_1_ receptor blockade by 36 pmol of SR141716A in the NTS on BRS for control of HR in SD rats. This is in line with previous observations by others that NTS microinjection of SR141716A alone in dogs did not affect BRS (Rademacher et al., 2003), nor did the CB_1_-selective antagonist AM281 by itself alter sympathetic nerve discharge in SD rats (Durakoglugil and Orer, 2008). Lack of responses may indicate minimal or absent tonic influence of the endocannabinoid system over baroreflex function in normotensive animals, or that baseline CB_1_ receptor modulation of the baroreflex in SD rats reflects balanced effects on GABA and glutamate release (Seagard et al., 2004; Chen et al., 2010). In fact, glutamate levels are significantly higher in the dorsal medulla of ASrAOGEN rats relative to (mRen2)27 rats as measured by proton magnetic resonance spectroscopy (Garcia-Espinosa et al., 2010). It is also possible that this reflects a compensatory balance of additional factors in the NTS, such as endovanilloids (Derbenev et al., 2006), leptin (Arnold et al., 2009), serotonin (Nalivaiko and Sgoifo, 2009), and nonesterified fatty acids (Shaltout and Abdel-Rahman, 2005) that may potentially influence baroreflex function. Although medullary endocannabinoids may modulate BRS through actions at transient receptor potential vanilloid type 1 channels in the NTS or dorsal motor nucleus (Anwar and Derbenev, 2013), our studies emphasize the contribution of CB_1_ receptors in baroreflex modulation, because SR141716A is a selective CB_1_ receptor antagonist that is not reported to interact with other receptors, including CB_2_ receptors (Rinaldi-Carmona et al., 1995).

Measurements of spontaneous BRS obtained by spectral analysis of the AP wave before and after NTS CB_1_ receptor blockade agree with the results of our evoked BRS measurements using the phenylephrine ramp method, providing additional evidence for altered CB_1_ receptor tone in the NTS of (mRen2)27 and ASrAOGEN rats. Analysis of the individual sympathetic (Seq Down) and parasympathetic (Seq Up) time sequences of sBRS revealed significant alterations only to Seq Up in the transgenic animals following administration of SR141716A, suggesting that NTS CB_1_ receptors may preferentially modulate BRS in response to increases in AP since the up-sequence is an accepted measure for tonic cardiac parasympathetic control (Wang et al., 2004). BRS values obtained by the spontaneous as compared with evoked methods reflect changes over a much smaller range (beat-to-beat) in a closed-loop model. Despite differences in the two methods, a highly significant correlation exists between them (Shaltout and Abdel-Rahman, 2005), especially with vagal components, as illustrated in this study and a recent study in older SD rats, which also found comparable results between the evoked and spectral analysis methods (Schaich et al., 2015). Furthermore, the absence of alterations in chemosensitive vagal fiber responses supports the specificity of SR141716A actions on BRS because these responses are mediated by chemoreceptor fibers that converge with baroreceptor inputs within the NTS (Paton, 1998).

We found that 2-AG content was highest in dorsal brainstem of (mRen2)27 rats and lowest in ASrAOGEN rats, which may be a potential mechanism contributing to differential resting BRS among SD, (mRen2)27 and ASrAOGEN rats. Anandamide may also play an important role in this region, but its detection at 1000-fold lower levels without significant differences among strains suggests that 2-AG is likely the more functionally significant endocannabinoid with respect to CB_1_-mediated BRS modulation at the level of the NTS in the face of altered brain Ang II/Ang-(1-7) balance. We further observed a significantly lower expression of CB_1_ receptor mRNA in dorsal medullary tissue of ASrAOGEN rats relative to SD and (mRen2)27 rats. Together, these observations suggest differential regulation of the endocannabinoid system in dorsal medulla of (mRen2)27 and ASrAOGEN rats, respectively, paralleling the relative expression of RAS components in these animals. In (mRen2)27 rats, the higher dorsal medullary 2-AG levels may reflect increased production (Szekeres et al., 2012), increased activity of enzymes involved in its biosynthesis, such as diacylglycerol lipase, or downregulation of its primary degradative enzyme monoacylglycerol lipase (Di Marzo, 2008) as the principal drivers of upregulated CB_1_ receptor tone in this region. Our data are consistent with evidence for upregulated endocannabinoid tone that is widely reported in acute and chronic animal models of cardiovascular disease, including atherosclerosis and hypertension (Pacher et al., 2005; Sugamura et al., 2009; Naito et al., 2010).

Although dorsal medullary expression of CB_1_ receptor in (mRen2)27 rats did not differ from SD rats, enhanced sensitivity of the receptor cannot be ruled out as an additional mechanism for upregulated CB_1_ receptor tone in this strain because Ang II-induced increased expression of G_*i*_ protein isoforms is reported in SHR (Marcil et al., 1997) and one-kidney one-clip hypertension (Ge et al., 2006). It is also possible that an interaction between CB_1_ and Ang II type 1 (AT_1_) receptors enhances the pathogenic signaling of Ang II, as previously described (Rozenfeld et al., 2011). In ASrAOGEN rats, lower levels of 2-AG and CB_1_ receptor mRNA suggest a more general downregulation of the dorsal medulla endocannabinoid system that may contribute to the enhanced baseline BRS for control of HR in these animals. Whether the differential expression of the CB_1_ receptor and production of 2-AG in the dorsal brainstem of (mRen2)27 and ASrAOGEN rats is attributable to a direct interaction with the RAS or an indirect effect is currently unknown; however, transactivation of CB_1_ by AT_1_ receptors and direct stimulation of 2-AG production by Ang II has been observed *in vitro* (Turu et al., 2007, 2009). Although we detected CB_2_ receptor mRNA in the NTS, it is likely not involved in BRS modulation because previous studies in our laboratory with the non-selective cannabinoid receptor agonist CP55,940 show all effects on BRS in SD rats are completely blocked or reversed by the CB_1_ antagonist SR141716A (Schaich et al., 2011). It is also unlikely that the CB_1_-associated protein CRIP1a, believed to serve an autoinhibitory function for CB_1_ receptors (Niehaus et al., 2007), is a primary contributor to differential modulation of BRS because we did not detect significant differences in mRNA expression among the three rat strains.

We cannot exclude the possibility that the spread of the SR141716A injection may have accessed neighboring brainstem nuclei, such as the area postrema or the dmnX, for effects on BRS. However, microinjection of ^125^I-Sar-Thr Ang II at this volume was mostly confined to the NTS (Campagnole-Santos et al., 1988), and functional assessments showed that 50 nL of an AT1 receptor antagonist injected into the dmnX did not alter responses to NTS microinjection of Ang II (Fow et al., 1994). The injection of SR141716A accessed NTS neuronal cell bodies as well as presynaptic vagal afferents and other terminals for projecting pathways within the NTS, so it is not clear which elements mediate the effects on BRS. However, Ang II exerts actions on BRS at neuronal cell fibers, glial and vascular elements in the NTS (Barnes et al., 2003).

We also cannot exclude the possibility that weak inverse agonist properties of SR141716A are reflected in our present data (Pertwee, 2005). Maximal inhibition of constitutive G-protein activity by SR141716A ranged from 20 to 40% at micromolar concentrations depending on brain region in rats, although effects in brainstem were not assessed (Sim-Selley et al., 2001). However, it is unclear if this property of SR141716A yields functionally significant actions *in vivo*. For example, SR141716A by itself only slightly reduced neuronal discharge in rat locus coeruleus (Muntoni et al., 2006), and in the NTS of dogs SR141716A had no effect on baseline AP or resting sympathetic BRS (Rademacher et al., 2003), fully consistent with our present data in SD rats. Potential inverse agonism effects notwithstanding, the increased levels of 2-AG we detected in dorsal brainstem of (mRen2)27 rats are consistent with a competitive antagonist effect of SR141716A in NTS to improve BRS, while the impairments to BRS in ASrAOGEN rats by SR141716A are too large to attribute to an inverse agonist effect.

We investigated changes in cardiovagal BRS, MAP and HR in response to acute, site-specific CB_1_ receptor blockade in rats with up- or downregulated brain RAS expression. Our results support our hypothesis for differential expression of brain endocannabinoid system components paralleling the brain RAS expression in these animals. The novel finding that NTS CB_1_ receptor blockade produces opposite effects in rats over- or under-expressing components of the brain RAS may have implications for understanding the mechanisms of autonomic reflex control of HR and blood pressure in pathological conditions that are in part dependent on an activated brain RAS, such as hypertension. In fact, chronic systemic treatment with SR141716 lowered blood pressure and improved BRS in transgenic (mRen2)27 rats (Schaich et al., 2014) and the present data implicate the NTS as at least one brain site contributing to these effects. Chronic selective peripheral and central CB_1_ receptor blockade is required to further evaluate the role of endocannabinoid-mediated BRS impairments in pathologies associated with altered circulating, cerebrospinal fluid, or brain tissue RAS.

### Perspectives

The impairment of BRS for control of heart rate, an index of vagus nerve function, often precedes the onset of hypertension and stroke (Thayer and Lane, 2007) and is a common feature of cardiovascular risk factors, including obesity and aging (Thayer et al., 2010). Identifying factors that modulate baroreflex function is therefore important for understanding predisposition or progression of these negative conditions. The present data suggest an upregulated brain endocannabinoid system in Ang II-dependent hypertension associated with a deficiency in the counterbalancing actions of Ang-(1-7) may contribute to the impaired BRS typical of these conditions, and that blockade of CB_1_ receptors improves BRS. We observed a similar pattern in older SD rats with age-related impairments in BRS known to be associated with Ang-(1-7) deficiency with the NTS (Schaich et al., 2015). CB_1_ receptor blockade within the NTS restored BRS to levels similar to younger SD rats and this was associated with elevated 2-AG content in dorsomedial medulla of the older rats (Schaich et al., 2015). Therefore, CB_1_ receptor-mediated impairments in BRS resulting from increased endocannabinoid production may contribute to factors permissive in the development of elevated AP in populations with elevated Ang II and/or deficiencies in Ang-(1-7). The positive metabolic effects of chronic systemic CB_1_ receptor blockade in obese or diabetic humans and animals are well known (Van Gaal et al., 2005). The results of our present study suggest that chronic systemic administration of a CB_1_ receptor antagonist may have additional, positive autonomic or cardiovascular effects in hypertension accompanied by impaired metabolic function. Further understanding of the consequences and mechanisms of an upregulated endocannabinoid system in hypertension will be important for therapeutic targeting of all features of cardiovascular disease.

## Author contributions

All authors wrote, edited and approved the final submission of the manuscript. CS, AH, and DD conceived the project and designed experiments. CS, MG, and HS performed experiments and collected and analyzed data. MG, BT, PG, and AH developed analytical tools.

## Funding

This work was supported by the National Heart, Lung, and Blood Institute grant HL-51952, the National Institute on Drug Abuse grant DA-03690, and RTI International (Research Triangle Park, NC). Support from the Farley-Hudson Foundation (Jacksonville, NC) and the Hypertension and Vascular Research Center is gratefully acknowledged.

### Conflict of interest statement

The authors declare that the research was conducted in the absence of any commercial or financial relationships that could be construed as a potential conflict of interest.
