# Supplementary material for: Medullary Endocannabinoids Contribute to the Differential Resting Baroreflex Sensitivity in Rats with Altered Brain Renin-Angiotensin System Expression
Source: Front Physiol. 2016 Jun 9;7:207. doi: 10.3389/fphys.2016.00207 (PMC4899471; doi:10.3389/fphys.2016.00207)
Supplement: Supplementary file 1 [file DataSheet1.docx]

ONLINE SUPPLEMENT:

**Medullary endocannabinoids contribute to the differential resting baroreflex sensitivity in rats with altered brain renin-angiotensin system expression**

Chris L. Schaich,^1^ Megan Grabenauer,^1, 2^ Brian F. Thomas,^1, 2^ Hossam A. Shaltout,^1, 3^ Patricia E. Gallagher,^1^ Allyn C. Howlett,^1^ Debra I. Diz^1^

^1^Department of Physiology and Pharmacology; and Hypertension & Vascular Research Center, Wake Forest School of Medicine, Winston-Salem, NC, USA

^2^Analytical Chemistry and Pharmaceutics, RTI International, Research Triangle Park, NC, USA

^3^Department of Obstetrics and Gynecology, Wake Forest School of Medicine, Winston-Salem, NC, USA

Short title: Endocannabinoids and Baroreflex Sensitivity

Address for Correspondence:

Debra I. Diz

Hypertension & Vascular Research Center

Wake Forest School of Medicine

Medical Center Boulevard

Winston-Salem, NC 27157-1032

Tel: (336) 716-2150

Fax: (336) 716-2456

[ddiz@wakehealth.edu](mailto:ddiz@wakehealth.edu)

**Table S1.** Values of MAP, HR and BRS in Response to NTS Microinjection of Vehicle

| **Group** | **N** | | **MAP**  **(mmHg)** | **HR**  **(bpm)** | **BRS**  **(ms/mmHg)** |
| --- | --- | --- | --- | --- | --- |
| **Vehicle in SD rats**  Baseline  Values at 10 minutes | | 5 | 93 ± 2  92 ± 3 | 330 ± 12  319 ± 15 | 1.02 ± 0.12  1.02 ± 0.10 |
| **Vehicle in (mRen2)27 rats**  Baseline  Values at 10 minutes | | 3 | 96 ± 8  92 ± 4 | 294 ± 20  296 ± 9 | 0.44 ± 0.04^**^  0.45 ± 0.06^**^ |
| **Vehicle in ASrAOGEN rats**  Baseline  Values at 10 minutes | | 4 | 119 ± 8^#^  121 ± 8^#^ | 360 ± 16  358 ± 9 | 1.43 ± 0.09^*^  1.46 ± 0.14^*^ |

Values are mean ± SEM and represent MAP, HR and BRS values at baseline and 10 minutes after NTS microinjection of vehicle; N = number of animals; MAP = mean arterial pressure; HR = heart rate.

^*^P < 0.05 vs. SD rats; ^#^P < 0.01.

**Figure S1. NTS Injection Site Histology**

Photomicrography (5X magnification) of a 30 µm unstained rat medullary section (approximately -13.8 mm caudal to bregma) depicting a typical NTS microinjection site (0.4 mm rostral, 0.4 mm lateral to the calamus scriptorius [caudal tip of the area postrema] and 0.4 mm beneath the dorsal surface). AP = area postrema; C = central canal; DMX = dorsal motor nucleus of the vagus; NG = nucleus gracilis; NTS = solitary tract nucleus.


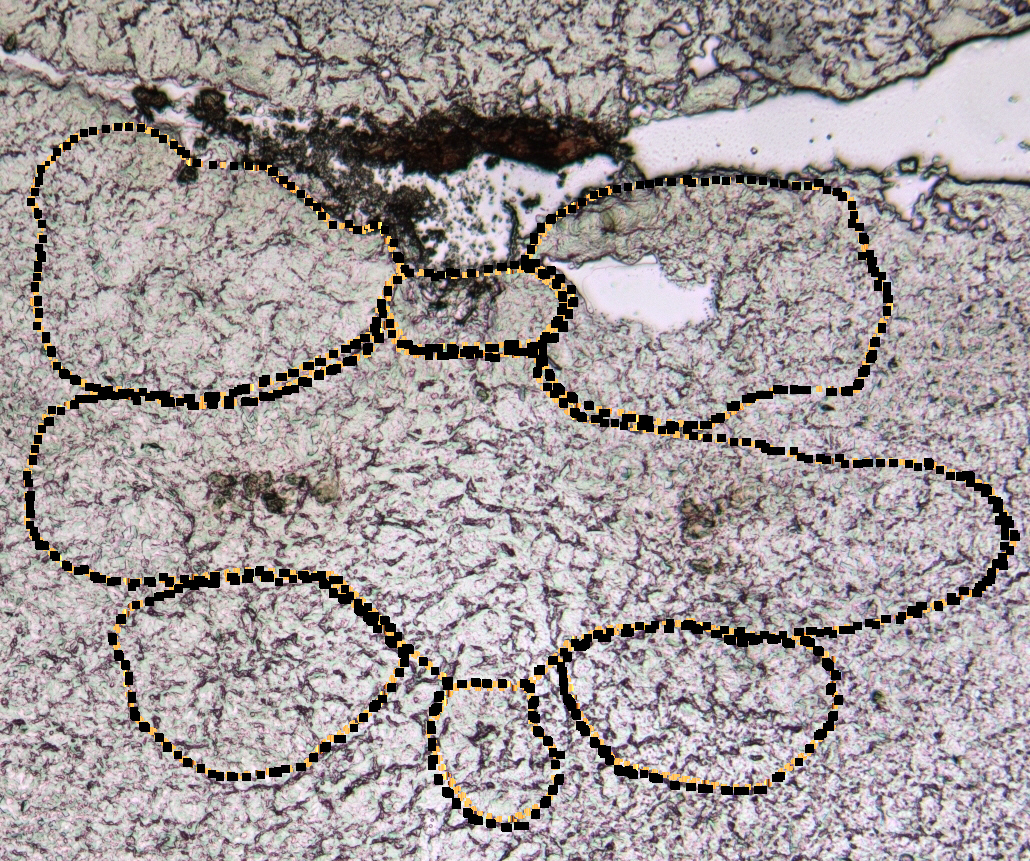


**NTS**

**NG**

**NG**

**dmnX**

**dmnX**

**C**

**Cerebellum**

**AP**

**Figure S2. Baseline BRS for Control of HR in SD, (mRen2)27 and ASrAOGEN Rats**

A, Pooled baseline BRS for control of HR in response to increases in AP evoked by PE prior to NTS microinjection of 120 nL of vehicle or SR141716A in anesthetized SD (n = 10), (mRen2)27 (n = 13) and ASrAOGEN (n = 11) rats. B, Scatterplot illustrates significantly different pooled baseline BRS reflex testing regression lines among ASrAOGEN (1.41 ± 0.15 ms/mmHg), SD (0.90 ± 0.09 ms/mmHg) and (mRen2)27 (0.48 ± 0.09 ms/mmHg) rats (P < 0.0001). ‡P < 0.001 vs. SD.

**Figure S3. Baseline BRS for Control of HR in SD, (mRen2)27 and ASrAOGEN Rat Treatment Groups**

There were no differences in baseline cardiac BRS measured prior to NTS microinjections of SR141716A (0.36 or 36 pmol) or vehicle among groups of anesthetized SD (n = 5), (mRen2)27 (n = 3-5) or ASrAOGEN (n = 3-4) rats receiving vehicle or various doses of SR141716A.

**Figure S4. Baseline MAP and HR of SD, (mRen2)24 and ASrAOGEN Rats**

A-B, Pooled baseline MAP (A) and HR (beats per minute [bpm]) (B) prior to NTS microinjection of 120 nL of vehicle or SR141716A (0.36 or 36 pmol) in anesthetized SD (n = 10), (mRen2)27 (n = 13) and ASrAOGEN (n = 11) rats. *P < 0.05 vs. SD; †P < 0.01 vs. SD; #P < 0.01 vs. (mRen2)27.

**Figure S5. MAP Responsiveness in SD, (mRen2)27 and ASrAOGEN Rats at Baseline and in Response to NTS Microinjection of SR141716A**

Changes in MAP in responsiveness to intravenous graded doses of PE were assessed in anesthetized SD, (mRen2)27 and ASrAOGEN rats at baseline and in response to NTS microinjection of 0.36 or 36 pmol of SR141716A. A, Baseline MAP responsiveness to 2 µg of PE was significantly greater in ASrAOGEN and (mRen2)27 rats compared to SD rats (P < 0.05). B, 36 pmol of SR141716A in SD rats significantly potentiated PE-induced increases in MAP at 10 and 60 minutes after NTS microinjection (P < 0.05; n = 5). C-D, There were no significant differences in PE-induced increases in MAP within groups of (mRen2)27 (C; n = 5 per group) or ASrAOGEN rats (D; n = 3-4 per group) 10 minutes after NTS microinjection.

**Figure S6. Effects of Chemoreflex Activation on MAP and HR Induced by Intravenous PBG in SD, (mRen2)27 and ASrAOGEN Rats**

No change in depressor or bradycardic responses to cardiac chemosensitive vagal fiber activation following NTS microinjection of SR141716A in SD, (mRen2)27 or ASrAOGEN rats (n = 3-5 all treatment groups; pooled baselines are represented in figures).
